# Development and validation of prediction model for overall survival in patients with lymphoma: a prospective cohort study in China

**DOI:** 10.1186/s12911-023-02198-0

**Published:** 2023-07-17

**Authors:** Xiaosheng Li, Yue Chen, Anlong Sun, Ying Wang, Yao Liu, Haike Lei

**Affiliations:** 1 Chongqing Cancer Multi-omics Big Data Application Engineering Research Center, Chongqing University Cancer Hospital, Chongqing, 400030 China; 2 Chongqing Key Laboratory of Translational Research for Cancer Metastasis and Individualized Treatment, Chongqing University Cancer Hospital, Chongqing, 400030 China

**Keywords:** Lymphoma patients, Prognosis, Survival, Prediction model

## Abstract

**Objective:**

The survival of patients with lymphoma varies greatly among individuals and were affected by various factors. The aim of this study was to develop and validate a prognostic model for predicting overall survival (OS) in patients with lymphoma.

**Methods:**

We conducted a prospective longitudinal cohort study in China between January 2014 and December 2018 (n = 1,594). After obtaining the follow-up data, we randomly split the cohort into the training cohort (n = 1,116) and the validation cohort (n = 478). The least absolute shrinkage and selection operator (LASSO) regression analysis was used to select the predictors of the model. Cox stepwise regression analysis was used to identify independent prognostic factors, which were finally displayed as static nomogram and web-based dynamic nomogram. We calculated the concordance index(C-index) to describe how the predicted survival of objectively confirmed prognosis. The calibration plot is used to evaluate the prediction accuracy and discrimination ability of the model. Net reclassification index (NRI) and decision curve analysis (DCA) curves were also used to evaluate the prediction ability and net benefit of the model.

**Results:**

Nine variables in the training cohort were considered to be independent risk factors for patients with lymphoma in the final model: age, Ann Arbor Stage, pathologic type, B symptoms, chemotherapy, targeted therapy, lactate dehydrogenase (LDH), β2-microglobulin and C-reactive protein (CRP). The C-indices of OS were 0.749 (95% CI, 0.729–0.769) in the training cohort and 0.731 (95% CI, 0.762–0.700) in the validation cohort. A good agreement between prediction by nomogram and actual observation was shown in the calibration curve for the probability of survival in both the training cohort and validation cohorts. The areas under curve (AUC) of the area under the receiver operating characteristic (ROC) curves for 1-year, 3-year, and 5-year OS were 0.813, 0.800, and 0.762, respectively, in the training cohort, and 0.802, 0.768, and 0.721, respectively, in the validation cohort. Compared with the Ann Arbor Stage system, NRI and DCA showed that the model had a higher predictive capacity and net benefit.

**Conclusion:**

The prediction models reliably estimate the outcome of patients with lymphoma. The model had high discrimination and calibration, which provided a simple and reliable tool for the survival prediction of the patients, and it might help patients benefit from personalized intervention.

## Introduction

Lymphomas are the most common hematologic malignancies, which originate from neoplastic clones of B, T, or natural killer (NK) cells. They are traditionally classified roughly into Hodgkin lymphoma (HL) and non–Hodgkin lymphoma (NHL), and HL only accounts for about 10% of all lymphomas [1, 2]. Although lymphoma is not a common cause of death worldwide, it still causes a large number of deaths. According to the systematic analysis of GLOBOCAN 2018, produced by the International Agency for Research on Cancer, lymphoma accounted for 2.9% of the 9.6 million cancer deaths worldwide in 2018, including 0.3% of deaths due to HL, 2.6% due to NHL [3]. The incidence and mortality of lymphoma increased compared to the statistics of GLOBOCAN 2012 [4]. It was estimated that lymphoid neoplasms accounted for 2.1% (88,200 new cases) of all new cancer cases and 1.9% (52,100 deaths) of all cancer deaths in China in 2015 [5]. Moreover, during the period 2004–2016, the mortality of lymphoma and myeloma increased by 4.5% annually [6]. Due to the high morbidity and mortality of lymphoma, it is necessary to provide a prediction tool to assess lymphoma patients’ prognosis.

The International Prognostic Index (IPI) is commonly used to evaluate the prognosis of lymphoma. IPI includes age, Ann Arbor stage, number/sites of involvement, patients’ performance status (PS), and serum lactate dehydrogenase (LDH) [7]. Moreover, various other factors, such as sex, pathologic type, treatment method, and β2-microglobulin may affect the overall survival (OS) of patients with lymphoma. Hence, a novel more accurate predictive model is still needed to assess the prognosis and to improve treatment strategies.

## Materials and methods

### Data source

This is a prospective cohort study based on the Chongqing University Cancer Hospital tumor database platform, which collected all patients with lymphoma newly diagnosed in the hospital since 2013. We selected patients admitted from January 1, 2013 to December 31, 2018, and prospectively collected demographics (sex, age at diagnosis, ethnic, medical insurance), clinical characteristics (Ann Arbor Stage, pathological type, and B symptoms), treatment methods (surgery, radiotherapy, chemotherapy, targeted therapy, and immunotherapy), laboratory indicators (LDH, β2-microglobulin, platelet, lymphocyte, albumin/globulin ratio, C-reactive protein), and follow-up information. Both radiotherapy and conservative treatment of traditional Chinese medicine were excluded from the data collection process because there were fewer data, much missing and difficult to analyze.

### Inclusion and exclusion criteria

**Fig. 1:**
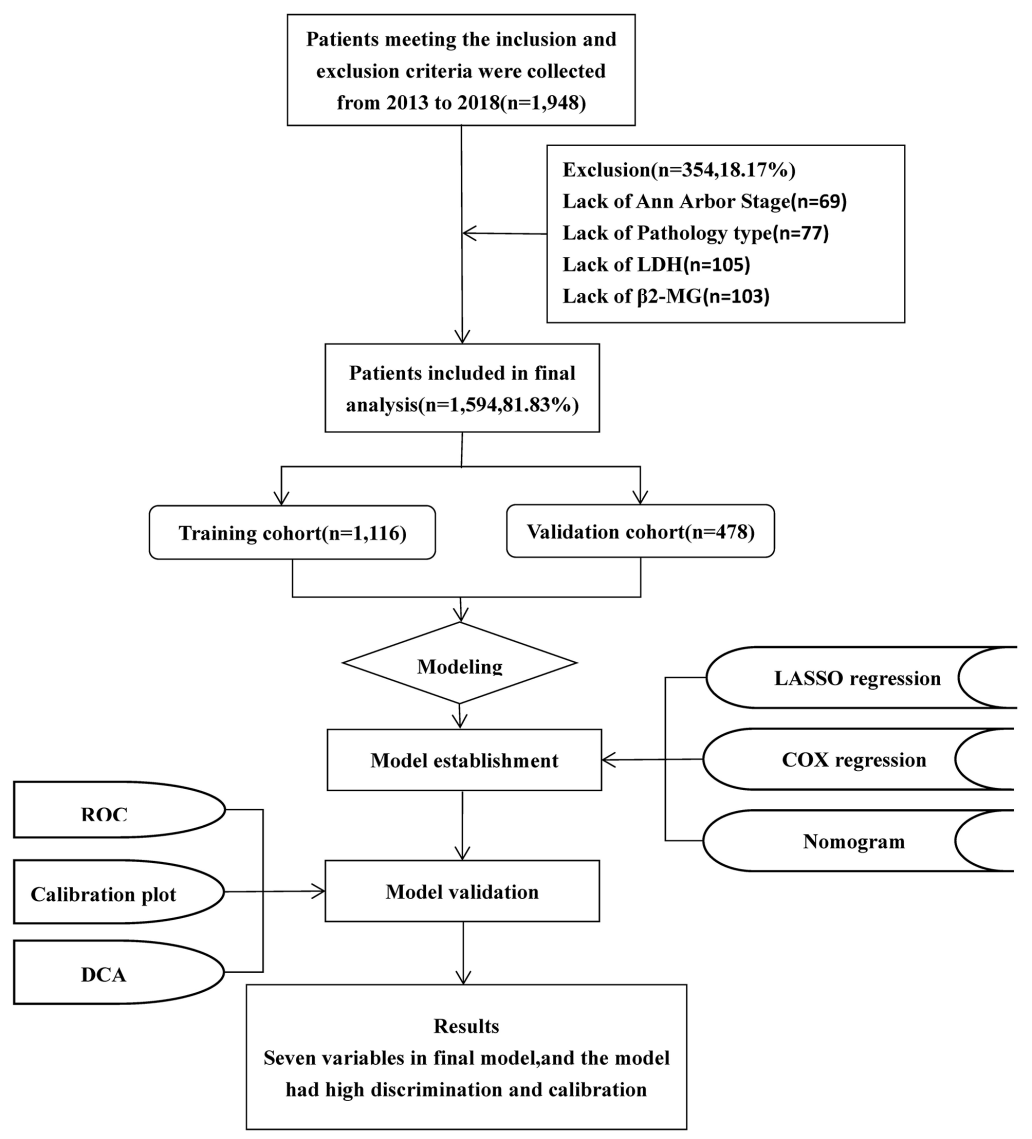
Flow diagram of study design

The inclusion criteria of this study were as follows: (1) age ≥ 18 years; (2) a record of at least one time of hospitalization; (3) with a new diagnosis of lymphoma by pathology (according to ICD-O-3 oncology codes); (4) no history of other types of malignancy; (5) major clinical treatment was completed at the hospital; (6) primary indicators and follow-up data were available. We excluded patients died within 48 h after admission or without any valid follow-up records. The flowchart of the study is provided in Fig. 1. The present study was performed according to the guidelines of the Declaration of Helsinki and was approved by the Ethics Committee of The Chongqing University Cancer Hospital. Written informed consent was obtained from all subjects.

### Laboratory tests

Blood samples were drawn from the antecubital vein and stored in vacuum tubes containing EDTA (Ethylene Diamine Tetraacetic Acid), and all the blood tests were done at the laboratory of the Chongqing University Cancer Hospital. The serum prognostic markers under investigation were LDH, β2-microglobulin, platelet count, lymphocyte count, albumin/globulin ratio, and C-reactive protein (CRP). According to the kit manufacturer, the upper normal limits of serum LDH, β2-microglobulin, platelet count, and CRP were 245U/L, 2.5 mg/L,300*10^9^/L, and 10 mg/L, respectively.

### Outcomes and follow-up

The primary outcome was the probability of 1-year,3-year, and 5-year OS. We defined OS as the time from the first diagnosis to death, loss to follow-up, or the last follow-up. All subjects were followed up every 3–6 months for the first 2 years of diagnosis, and then annually thereafter until death. The study was censored on April 2022, and the follow-up period was in months. The median survival time of patients in this study was 86.70 months (95% CI: 73.17–88.94). We used a combination of active and passive follow-up to obtain patient survival outcomes. Active follow-up collected patient prognostic data via the phone. Passive follow-up tracked the prognosis of patients by matching their recent outpatient or inpatient information in the hospital information system.

### Construction and validation of clinical prediction model

Nomogram is an integrative graphical calculation or algorithm that incorporates biological and clinical variables, which is the most widely used to predict individual prognosis in clinical investigations currently [8, 9]. It has become an important instrument for clinical decision-making and risk stratification in oncology [10]. The present study constructed and validated a nomogram to predict OS of lymphoma patients.

We used the data to develop and validate a clinical model to evaluate the prognosis of lymphoma patients. We randomly divided the patients into two cohorts, the training cohort and validation cohorts, which included 70% and 30% of all patients, respectively. Variable selection of the training cohort was firstly performed by the LASSO Cox regression model. Dummies were created for categorical variables. Cross-validation was used to confirm suitable tuning parameters (λ) for LASSO regression, and the most significant variables were finally selected by LASSO. Then these features were used for multiple Cox proportional hazard analysis to confirm the significant predictors of OS along with hazard ratios (HRs) and corresponding 95% confidence intervals (CIs) in the training cohort. Based on the results of the Cox regression model, a nomogram was established for predicting the probability of 1-, 3-, and 5-year OS. The nomogram performance for predicting survival outcomes was further evaluated with discrimination and calibration in the validation cohort. The calibration curve was drawn to evaluate the prediction accuracy and discrimination ability of the model, and the ROC curve was drawn to verify the generalization ability of the model. The NRI and IDI of the nomogram were also calculated and used to evaluate the degree of improvement in accuracy and predictive ability compared with those of the model of the Ann Arbor Stage system [11]. Furthermore, the DCA was carried out to evaluate the potential clinical value of prediction models [12]. Nomograms were subjected to 1,000 bootstraps resamples for validation in the training cohort and validation cohort, respectively.

### Statistical analysis

Demographic and clinical variables were compared between the training and validation cohorts using the Pearson Chi-square test for categorical variables. The optimal cutoff values for age, lymphocyte count and albumin/globulin ratiowere calculated by X-tile software (Yale University, New Haven, CT, USA) [13]. Continuous variables such as age, LDH, β2-microglobulin, platelet count, lymphocyte count, albumin/globulin ratio,and CRP were converted to categorical variables using identified cut points. LASSO regression and multivariate Cox regression analysis were performed to select the significant features. R software version 4.1.0 (Institute for Statistics and Mathematics, Vienna, Austria) was used for the above statistical analysis, and statistical significance would be observed when the P value was below 0.05 in a two-tailed test. The “shiny” and “DynNom” packages were used to develop an online calculator based on the nomogram (https://www.shinyapps.io/) for individually and dynamically predicting patient survival rates.

## Results

### Baseline characteristics

**Fig. 2:**
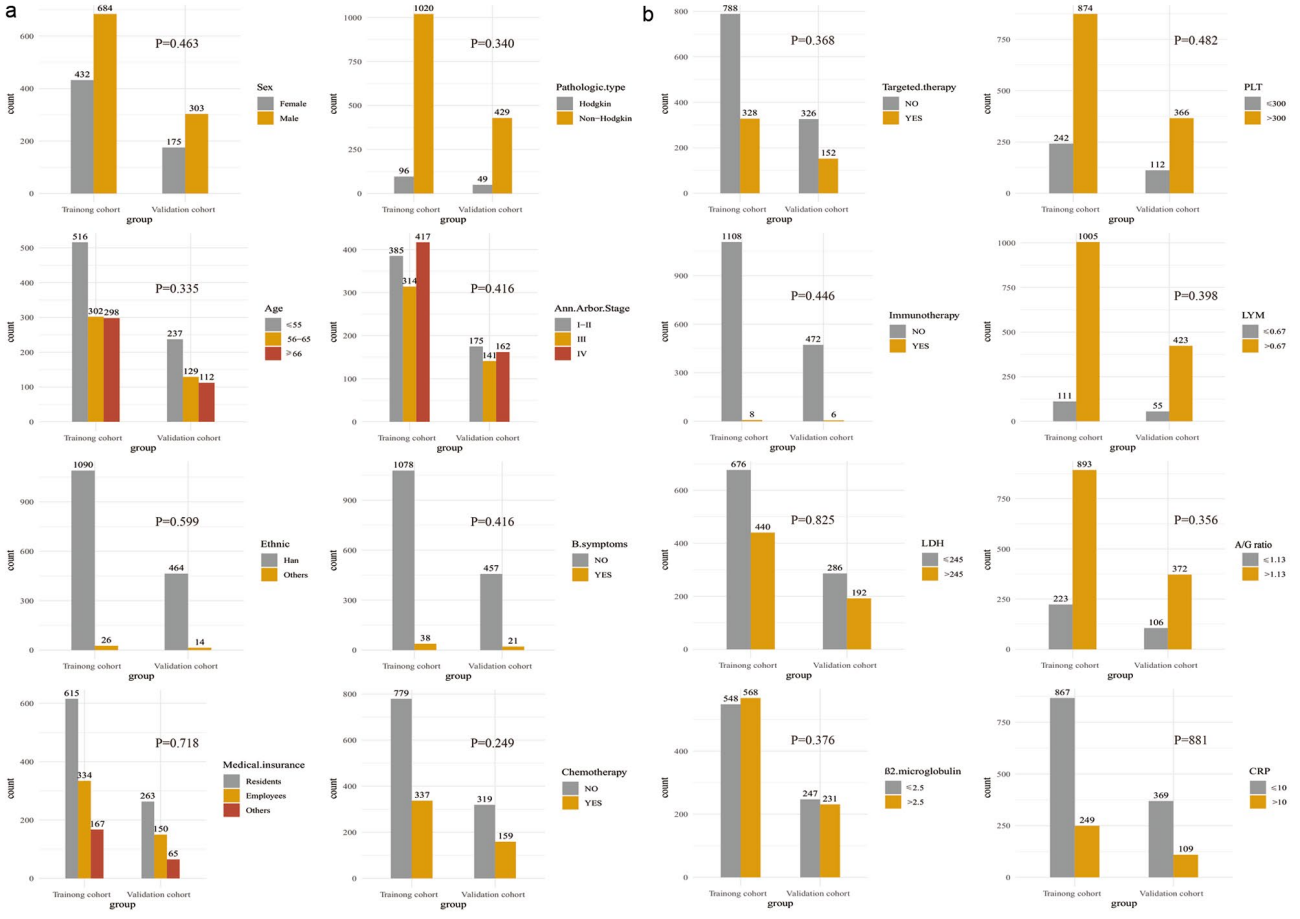
(**A**) Clinical characteristics of lymphoma patients in the training and validation cohorts. (**B**) Clinical characteristics of lymphoma patients in the training and validation cohorts(LDH, lactate dehydrogenase; PLT, Platelet count; LYM, Lymphocyte count; A/G ratio, albumin/globulin ratio; CRP, C-reactive protein)

A total of 1594 patients with integrated information were incorporated in the study, which was randomly divided into the training (n = 1116) and validation (n = 478) cohorts at a ratio of 7:3. There were 987(61.92%) male patients and 607(38.08%) female patients, and 47.24% of patients were younger than 55 years old. The majority of patients had NHL (90.90%). The demographic, clinicopathological, and treatment characteristics of the total cohort are listed in Fig. 2A and B, and there were no statistically significant differences between the training and validation cohorts. After a median follow-up of 37.50 months in the training cohort and 37.50 months in the validation cohort, 561 patients (50.30%) in the training cohort and 245 (51.30%) patients in the validation cohort died.

### Variable selection

**Fig. 3:**
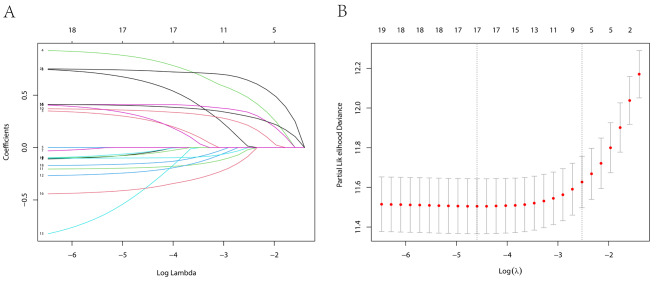
Variable selection by LASSO COX regression model. A coefficient profile plot was produced against the log(lambda) sequence (**A**). 17 variables with nonzero coefficients were selected by optimal lambda. By verifying the optimal parameter (lambda) in the LASSO model, the partial likelihood deviance (binomial deviance) curve was plotted versus log(lambda) and dotted vertical lines were drawn based on 1 standard error criteria (**B**)

We identified the variables of the nomogram in two steps. Firstly, based on each feature for variable selection, the LASSO regression algorithm was applied in the training cohort. The most appropriate tuning parameter λ for LASSO regression was 0.010 when the partial likelihood binomial deviance reached its minimum value (Fig. 3A). And17 variables with nonzero coefficients were selected by optimal lambda (Fig. 3B). Additionally, pathologic type and targeted therapy, which might affect prognosis, were included. Secondly, the multivariate Cox regression was used to analyze the remaining 9 features. Age (HR 2.440, 95% CI 1.994–2.984, P < 0.001), Ann Arbor Stage (HR 1.551, 95% CI 1.252–1.923, P < 0.001), pathologic type (HR 2.138, 95% CI 1.435–3.187, P < 0.001), B symptoms(HR 1.444, 95% CI 0.969–2.152, P = 0.071), chemotherapy (HR 0.778, 95% CI 0.623–0.971, P = 0.026), targeted therapy (HR 0.756, 95% CI 0.612–0.936, P = 0.010), LDH (HR 1.639, 95% CI 1.365–1.967, P < 0.001), β2-microglobulin (HR 1.577, 95% CI 1.298–1.917, P < 0.001), and CRP (HR 2.196, 95% CI 1.807–2.669, P < 0.001) were independent predictors for OS in patients with lymphoma (Table 1).

**Table 1:** Univariate and multivariate Cox regression analysis predicting OS in patients with lymphoma in training cohort

Variables	Univariate analysis		Multivariate analysis	
	HR (95%CI)	P-value	HR (95%CI)	P-value
Sex				
Female	1(ref.)			
Male	1.17(0.986–1.39)	0.072		
Age				
≤55	1(ref.)		1(ref.)	
56–65	1.46(1.19–1.81)	< 0.001	1.400(1.133–1.732)	0.002
≥66	3.03(2.49–3.69)	< 0.001	2.440(1.994–2.984)	< 0.001
Medical insurance				
Residents	1(ref.)			
Employees	0.938(0.774–1.14)	0.516		
Others	1.12(0.89–1.41)	0.333		
Pathologic type				
Hodgkin	1(ref.)		1(ref.)	
Non- Hodgkin	2.2(1.5–3.21)	< 0.001	2.138(1.435–3.187)	< 0.001
B symptoms	1.53(1.04–2.25)	0.031	1.444(0.969–2.152)	0.071
Ann Arbor Stage				
I-II	1(ref.)		1(ref.)	
III	1.42(1.13–1.78)	0.003	1.039(0.822–1.315)	0.748
IV	2.39(1.95–2.93)	< 0.001	1.551(1.252–1.923)	< 0.001
Chemotherapy	0.585(0.476–0.718)	< 0.001	0.778(0.623–0.971)	0.026
Targeted therapy	0.656(0.54–0.797)	< 0.001	0.756(0.612–0.936)	0.010
Immunotherapy	0.17(0.024–1.21)	0.077		
LDH > 245U/L	2.47(2.09–2.91)	< 0.001	1.639(1.365–1.967)	< 0.001
β2-microglobulin > 2.5 mg/L	2.72(2.28–3.24)	< 0.001	1.577(1.298–1.917)	< 0.001
CRP > 10 mg/L	3.47(2.9–4.14)	< 0.001	2.196(1.807–2.669)	< 0.001

HR, hazard ratios; CI, Confidence Interval

### Construction and validation of the Nomogram

**Fig. 4:**
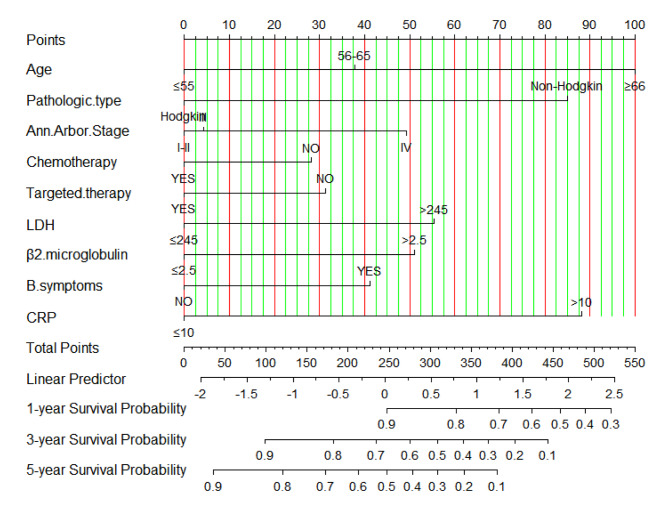
Nomogram for predicting 1-, 3- and 5-year overall survival of patients with lymphoma

The significant independent prognostic factors of OS for lymphoma patients were used to construct the nomogram. Although B symptoms did not reach statistical significance in the COX regression analysis, it was often associated with poor prognosis. Hence, we included it into the nomogram. In the nomogram, each risk factor was assigned a score, which can be obtained from the ruler above and superimposed on the ruler below to predict 1-, 3-, and 5-year OS (Fig. 4). Additionally, we developed an online calculator based on the nomogram (https://cqcgcp.shinyapps.io/DynNomapp/) to predict long-term OS in patients with lymphoma. As an example to better explain the model, if a 68-year-old Hodgkin patient with stage IV, LDH of 268 U/L, β2-microglobulin of 2.8 mg/L, and CRP of 10 mg/L, without B symptoms, chemotherapy nor targeted therapy, the probability of 5-year OS rate is estimated to be 28.0%.

**Fig. 5:**
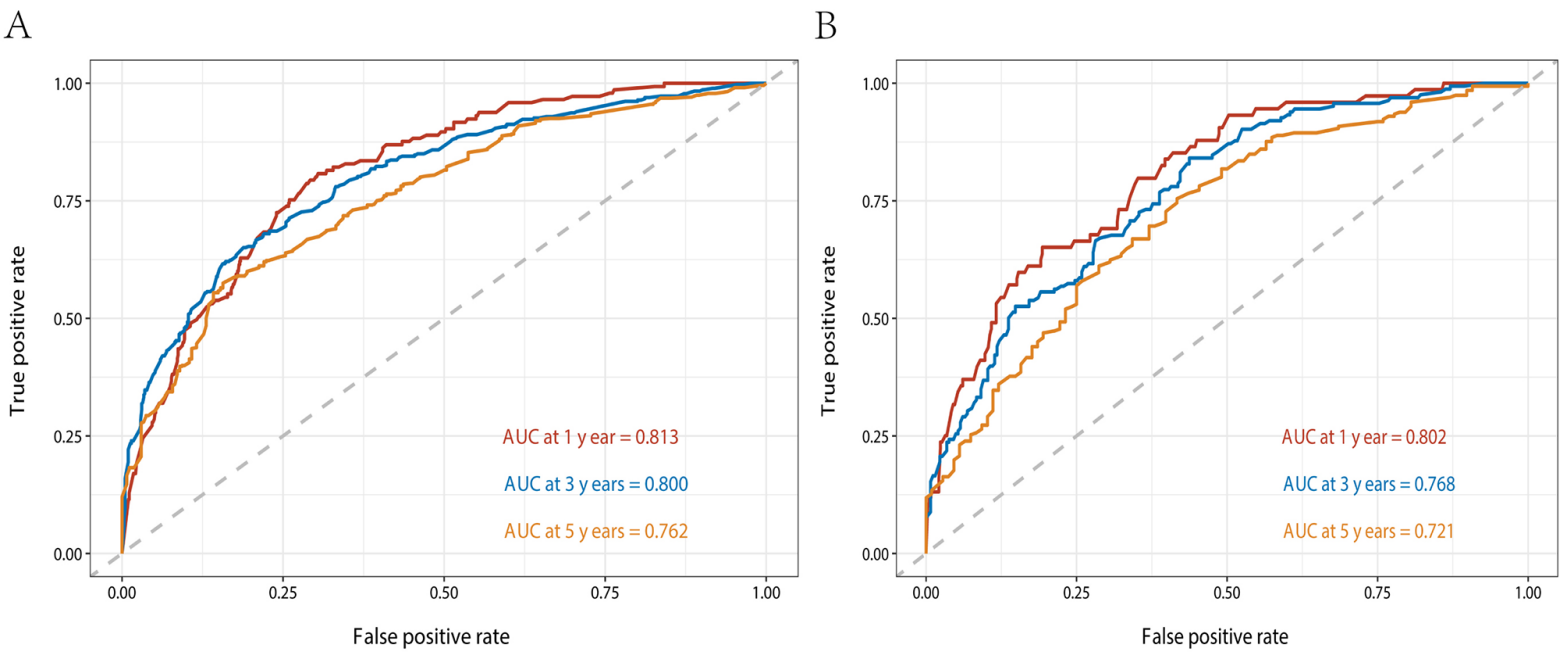
ROC curves of the nomogram for 1-, 3- and 5-year overall survival prediction in the training cohort (**A**) and validation cohort (**B**)

**Fig. 6:**
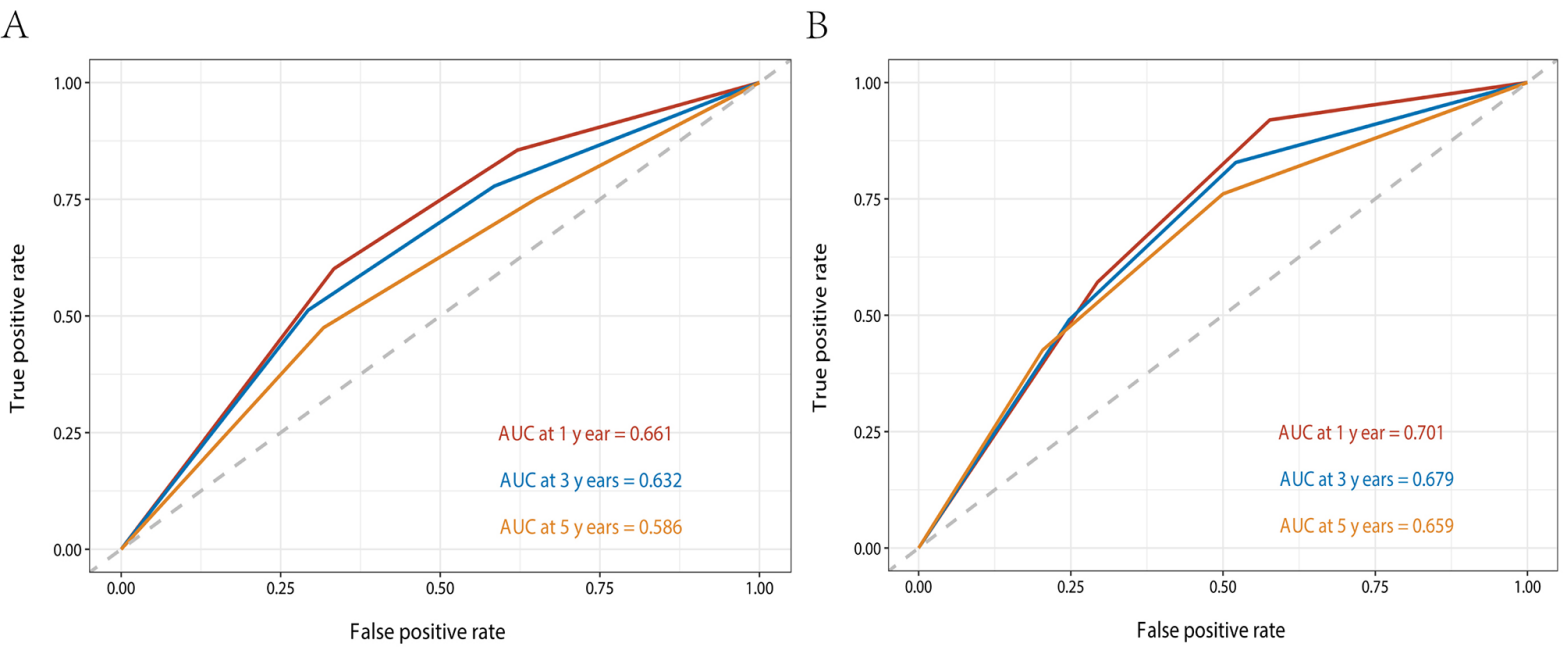
ROC curves of the Ann Arbor Stage for 1-, 3- and 5-year overall survival prediction in the training cohort (**A**) and validation cohort (**B**)

**Fig. 7:**
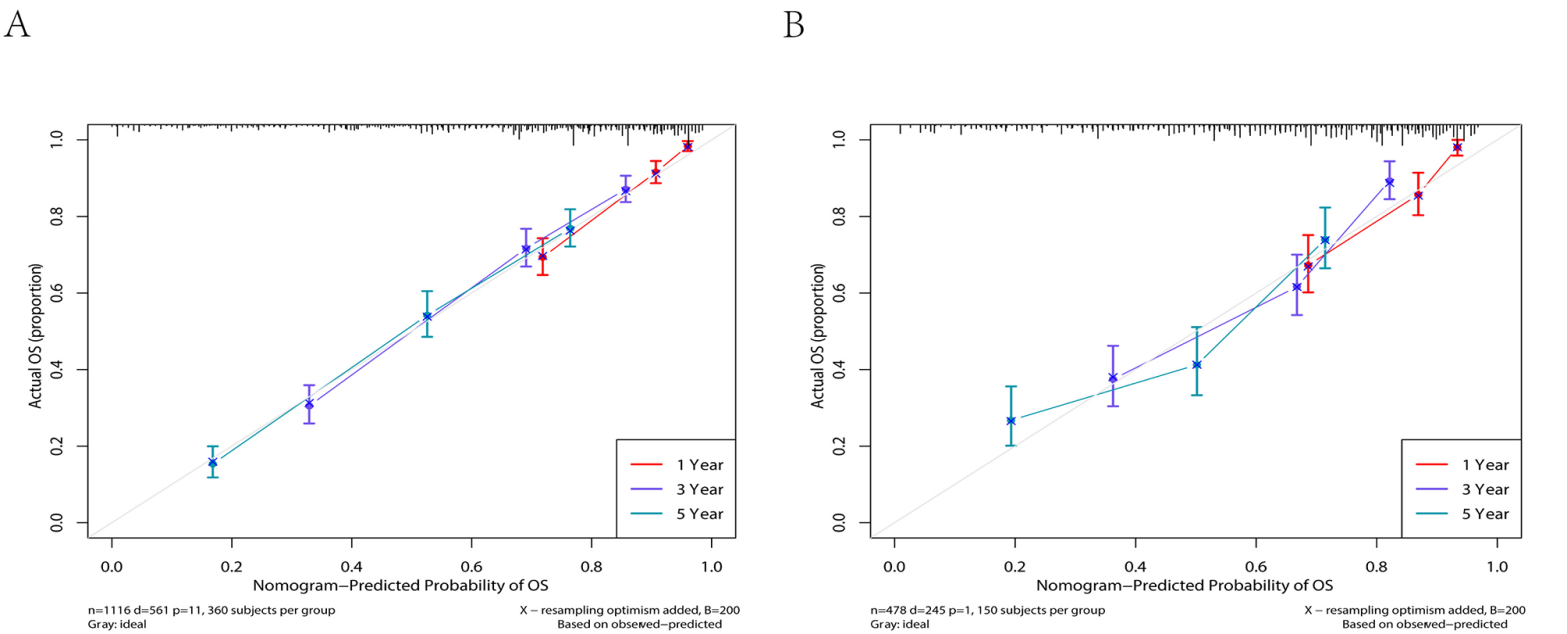
Calibration plot of the nomogram for 1-, 3- and 5-year overall survival in the training cohort (**A**) and validation cohort (**B**)

**Fig. 8:**
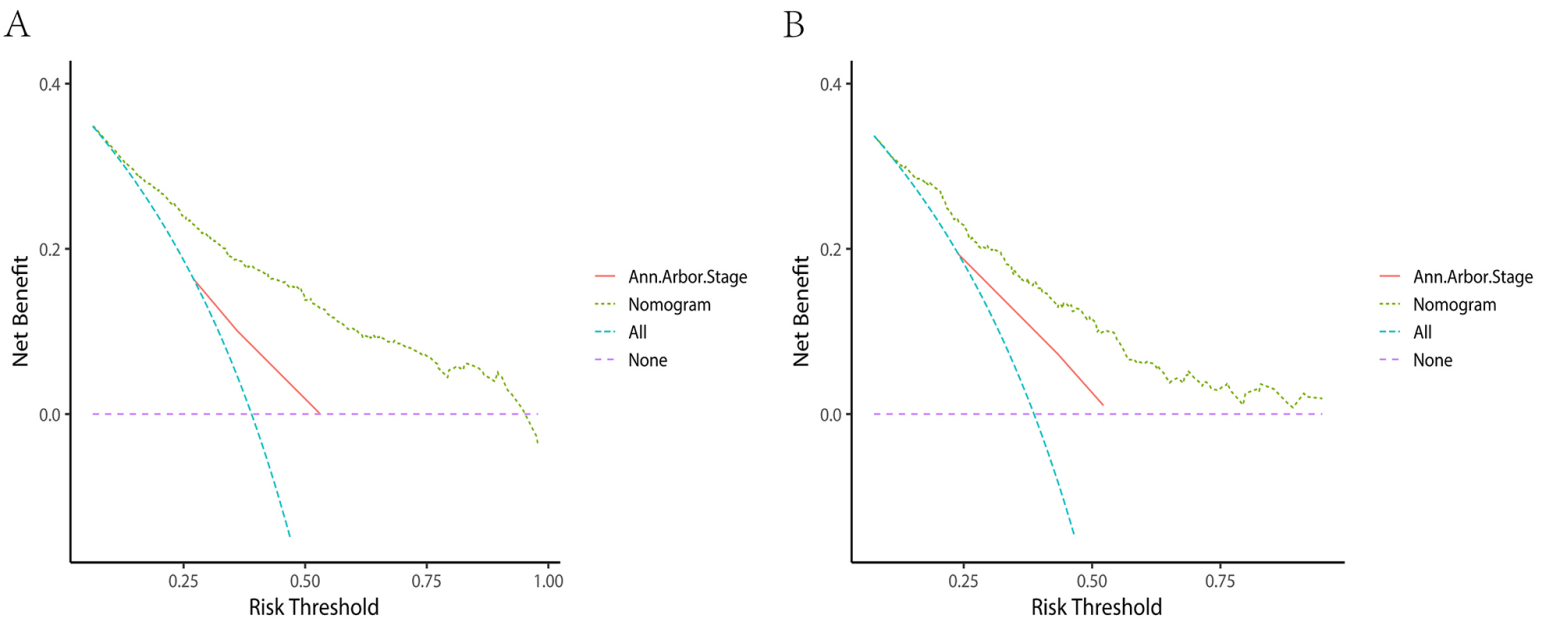
Decision curve analysis for the nomogram’s ability to predict 5-year overall survival in lymphoma patients in the training cohort (**A**) and validation cohort(**B**)

The C-indices of the nomogram for predicting the OS of lymphoma patients were calculated to be 0.749 (95% CI, 0.729–0.769) in the training cohort and 0.731 (95% CI, 0.762–0.700) in the validation cohort, indicating good discrimination. ROC values in the training cohort for predicting the 1-, 3-, and 5- year OS were 0.813, 0.800, and 0.762, respectively (Fig. 5A), and 0.802, 0.768, and 0.721, respectively, in the validation cohort (Fig. 5B). However, ROC curves of the Ann Arbor Stage for 1-, 3- and 5-year overall survival prediction were 0.661, 0.632, and 0.586 in the training cohort (Fig. 6A), 0.701, 0.679 and 0.659 in the validation cohort (Fig. 6B). The calibration curves for the 1-, 3- and 5- year OS showed good concordance between the predicted and observed probabilities by the nomogram both in the training (Fig. 7A) and validation cohorts (Fig. 7B). These results indicated the favorable survival predictive ability and accuracy of the model. The DCA analysis for the performance of the nomogram and Ann Arbor Stage in predicting 5-year OS in the training (Fig. 8A) and validation (Fig. 8B) cohorts showed that nomogram had higher net benefits and more accurate clinical outcome predictive values than those obtained using Ann Arbor Stage. Compared with the Ann Arbor Stage model, NRI, and IDI of the nomogram for the 5-year OS were 0.367(95%CI, 0.322–0.456) and 0.194(95%CI, 0.155–0.239) in the training cohort, 0.238(95%CI, 0.144–0.372) and 0.098(95%CI, 0.049–0.162) in the validation cohort. These results demonstrated that the nomogram was significant improvement in the prediction of 5-year OS in lymphoma patients.

**Fig. 9:**
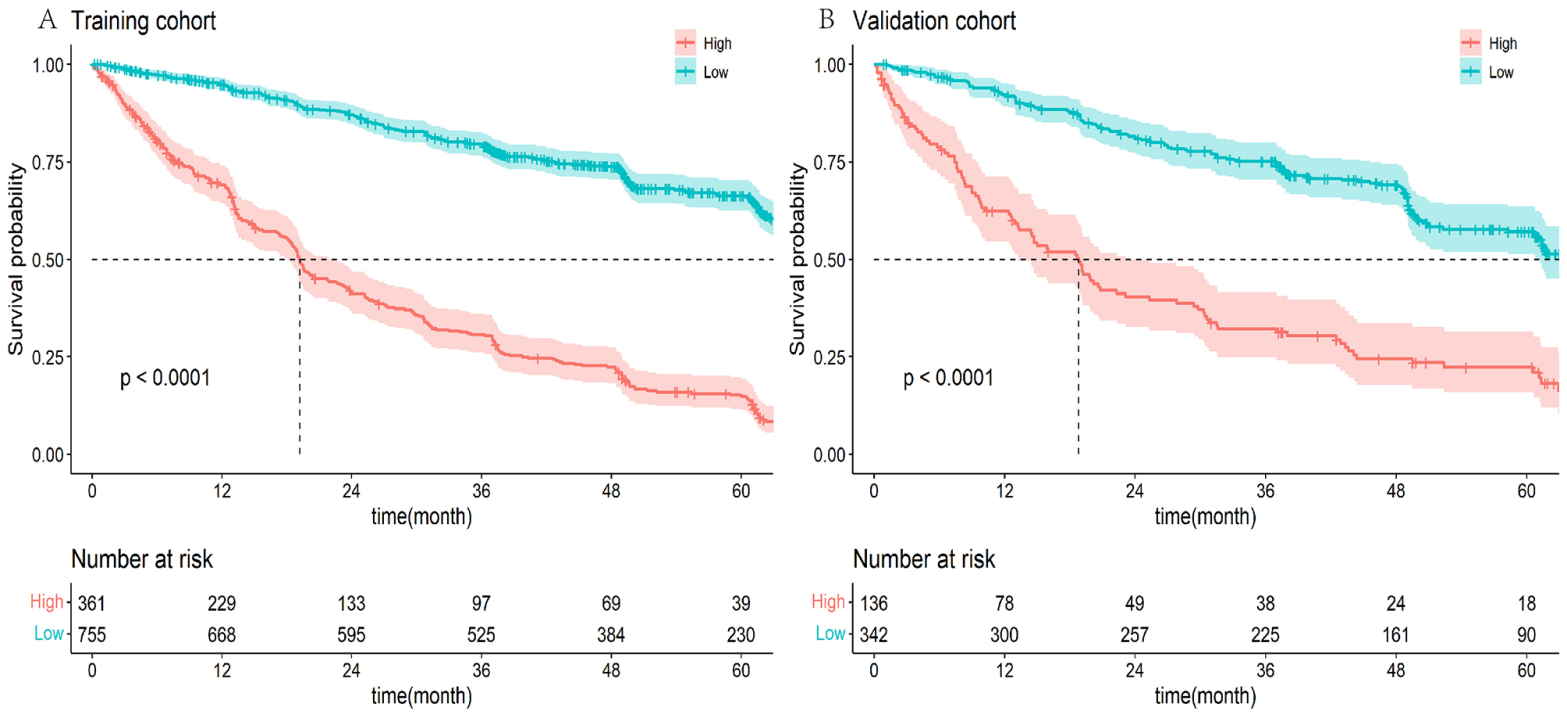
The nomogram distinguished the risk of lymphoma patients in the training cohort (**A**) and the validation cohort (**B**)

Based on the constructed model, we calculated the number of patients with high and low risk in the training set and validation set, as shown in Fig. 9. The results showed that the model of this study had good ability to identify high and low risks (P < 0.05).

## Discussion

A clinical prediction model with seven variables for the OS of lymphoma patients was developed in our study. Age, Ann Arbor Stage, pathologic type, B symptoms, chemotherapy, targeted therapy, LDH, β2-microglobulin, and CRP were included in the prognostic model, which were easily accessible in clinical practice, and improved the practicability of this model. The model had good calibration, discrimination, and predictive accuracy predictive for clinical outcomes and potential clinical decision-making value. Compared with the Ann Arbor stage, the nomogram showed a favorable level of predictive accuracy according to the AUC values. Furthermore, NRI and IDI values demonstrated that the proposed model could potentially be more beneficial than the Ann Arbor Stage system for predicting the OS of lymphoma patients. Several prognostic nomograms were previously developed for patients with lymphoma. Although some of these models included biomarkers, the sample was relatively small [14, 15]. And other large sample-size studies did not incorporate clinical biomarkers [16–19]. These nomograms might not be easily applied in daily clinical practice due to the need of more features to increase accuracy and practicality. In the present study, we regarded some biomarkers and clinicopathological parameters as research indicators and constructed a more practical nomogram. Additionally, the design of the online calculator based on the nomogram made it more convenient for clinicians to dynamically predict the OS rate of lymphoma patients at different time points. As the changing of OS rate, it would provide evidence for clinicians to carry out the continuous adjustment of their treatment strategies.

Age is used as a common predictive and prognostic factor in patients with lymphoma [19–21]. A previous study demonstrated that diffuse large B-cell lymphoma patients older than 65 years nearly had a 5-fold increased risk of death [19], which was similar to our results. It was reasonable that patients of advanced age had a higher mortality than younger patients due to the more comorbidities, decreased immunity, and increased therapy-related toxicity.

Ann Arbor Stage also was an independent risk factor for poor prognosis in patients with lymphoma, which was consistent with those in previous studies [18, 19]. The positive correlation between Ann Arbor Stage and mortality was in accordance with the development of disseminated disease.

Compared to Patients with HL, those with NHL have a worse prognosis [22]. Hence, the pathologic type as a separate prognostic index was included in the novel nomogram. And it suggested that the pathologic type should be considered when predicting the OS rate of lymphoma patients.

B symptoms was an independent prognostic factor of OS in lymphoma patients in the present study, which was consistent with some previous research [18, 19].

Different strategies such as chemotherapy, radiotherapy, targeted therapy, and immunotherapy have been used to treat lymphoma, which displays favorable clinical features and good prognosis [23]. Therefore, therapeutic factors play an important role in the individual survival of lymphoma patients. In our study, patients received chemotherapy or targeted therapy had significantly better prognoses than those who did not receive above-mentioned treatment. Although many patients received combined treatments, such as surgery combined with chemotherapy, radiotherapy combined with chemotherapy, and chemotherapy combined with targeted therapy, the Lasso regression analysis showed that chemotherapy and targeted therapy were independent factors for survival. These results would justify the active selection of treatment strategies.

LDH is a cytoplasmic isoenzyme catalyzing the reversible transformation of pyruvic acid to lactate in the terminal stage of the glycolytic enzymatic pathway, which is necessary for the survival of both normal and cancer cells. Cytoplasmic LDH can be released into the serum through disruption of cellular membranes, tumor lysis, and from other causes of cell and tissue damage. This has led to LDH emerged as a meaningful prognostic biomarker in neoplastic diseases. It is a negative correlation between elevated levels of LDH and the survival of lymphoma patients. A recent investigation showed that more than 3 times the normal LDH was an independent risk factors for OS of patients with diffuse large B-cell lymphoma [24]. Garcia et al. [25] found that the level of LDH > 320 U/L and age had prognostic influence on achieving complete remission (CR). This study demonstrated that elevated LDH was one of the most important prognostic factors in HL. Another research also showed that a high level of LDH was a potent negative prognostic variable in NHL in the autologous transplant setting [26, 27]. Eventually, the elevated level of LDH has been listed as a risk factor in IPI for NHL [7]. In accordance with previous studies, elevated level of LDH was proved to be associated with poor prognosis in lymphoma and included in the nomogram.

β2-microglobulin constitutes the light chain subunit of the human leukocyte antigen-I (HLA-I) and is synthesized by almost all nucleated cells, which is distributed on the cellular membrane [28]. The prognostic value of serum β2-microglobulin has been widely investigated in NHL and HL [29–31]. A recent retrospective study assessed the prognostic association between β2-microglobulin and diffuse large B-cell lymphoma in 621 patients in the rituximab era. It revealed that β2-microglobulin > 2.5 mg/L was an independent prognostic factor for survival in multivariate analysis [32]. Furthermore, the prognostic implication of serum β2-microglobulin in patients with mucosa-associated lymphoid tissue (MALT) lymphoma was documented in a large-scale retrospective study [31]. In our analysis, a high level of serum β2-microglobulin (> 2.5 mg/L) was independently associated with significantly worse OS. It highlighted the potential clinical value of incorporating serum β2-microglobulin into the prognostic nomogram for lymphoma.

CRP has been identified as a prognostic factor in various hematological malignancies, and higher CRP levels were associated with worse OS in patients with lymphoma. A recent study showed that elevated CRP was related to decreased 5-year OS in diffuse large B-cell lymphoma patients [33]. The relationship between CRP and long-term survival in extranidal natural killer (NK)/T-cell lymphoma was also investigated, which suggested CRP was an independent predictor of clinical outcome [34]. Our results were in line with previous research also demonstrating a prognostic value for CRP concentrations in lymphoma.

Our study has several limitations. Firstly, models were developed and validated in cohorts from a single medical center, which limits the generalization to other regions. Secondly, it is necessary to consummate an external validation of the predictive model. Thirdly, although the population was relatively large, the findings need to be confirmed by a larger and prospective cohort. Fourthly, the lymphoma was only divided into Hodgkin’s lymphoma (HL) and non-Hodgkin’s lymphoma (NHL) in the study, but the prognosis of different subtypes of NHL varies widely, and we hope that further studies will be conducted at a later stage for the different subtypes of NHL. Finally, this study has no imaging data for the time being. In the follow-up study, the imaging data will be further included in the analysis, which makes the integrity of the study stronger.

## Conclusion

In conclusion, we developed a predictive nomogram of OS in lymphoma patients with high discrimination and calibration. The application of the proposed novel model provided a simple and reliable tool for the survival prediction of the patients, and it might help patients benefit from personalized intervention.

## Data Availability

The datasets generated and/or analyzed during the current study are not publicly available to the privacy concerns of research committee but are available from the corresponding author on reasonable request.
